# Carbon monoxide-Releasing Molecule-2 (CORM-2) attenuates acute hepatic ischemia reperfusion injury in rats

**DOI:** 10.1186/1471-230X-10-42

**Published:** 2010-05-05

**Authors:** Yunwei Wei, Ping Chen, Marco de Bruyn, Weihui Zhang, Edwin Bremer, Wijnand Helfrich

**Affiliations:** 1 Third department of General Surgery, First Clinical Hospital Harbin, Harbin Medical University, Harbin 150001, Heilongjiang, China; 2 Department of Hepatobiliary Cancer Surgery, Tianjin Medical University Cancer Hospital and City Key Laboratory of Cancer Prevention and Therapy, Tianjin 300060, China; 3 Department of Surgery, Surgical Research Laboratories, University Medical Center Groningen, University of Groningen, Groningen, the Netherlands

## Abstract

**Background:**

Hepatic ischemia-reperfusion injury (I/Ri) is a serious complication occurring during liver surgery that may lead to liver failure. Hepatic I/Ri induces formation of reactive oxygen species, hepatocyte apoptosis, and release of pro-inflammatory cytokines, which together causes liver damage and organ dysfunction. A potential strategy to alleviate hepatic I/Ri is to exploit the potent anti-inflammatory and cytoprotective effects of carbon monoxide (CO) by application of so-called CO-releasing molecules (CORMs). Here, we assessed whether CO released from CORM-2 protects against hepatic I/Ri in a rat model.

**Methods:**

Forty male Wistar rats were randomly assigned into four groups (n = 10). Sham group underwent a sham operation and received saline. I/R group underwent hepatic I/R procedure by partial clamping of portal structures to the left and median lobes with a microvascular clip for 60 minutes, yielding ~70% hepatic ischemia and subsequently received saline. CORM-2 group underwent the same procedure and received 8 mg/kg of CORM-2 at time of reperfusion. iCORM-2 group underwent the same procedure and received iCORM-2 (8 mg/kg), which does not release CO. Therapeutic effects of CORM-2 on hepatic I/Ri was assessed by measuring serum damage markers AST and ALT, liver histology score, TUNEL-scoring of apoptotic cells, NFkB-activity in nuclear liver extracts, serum levels of pro-inflammatory cytokines TNF-α and IL-6, and hepatic neutrophil infiltration.

**Results:**

A single systemic infusion with CORM-2 protected the liver from I/Ri as evidenced by a reduction in serum AST/ALT levels and an improved liver histology score. Treatment with CORM-2 also up-regulated expression of the anti-apoptotic protein Bcl-2, down-regulated caspase-3 activation, and significantly reduced the levels of apoptosis after I/Ri. Furthermore, treatment with CORM-2 significantly inhibited the activity of the pro-inflammatory transcription factor NF-κB as measured in nuclear extracts of liver homogenates. Moreover, CORM-2 treatment resulted in reduced serum levels of pro-inflammatory cytokines TNF-α and IL-6 and down-regulation of the adhesion molecule ICAM-1 in the endothelial cells of liver. In line with these findings, CORM-2 treatment reduced the accumulation of neutrophils in the liver upon I/Ri. Similar treatment with an inactive variant of CORM-2 (iCORM-2) did not have any beneficial effect on the extent of liver I/Ri.

**Conclusions:**

CORM-2 treatment at the time of reperfusion had several distinct beneficial effects on severity of hepatic I/Ri that may be of therapeutic value for the prevention of tissue damage as a result of I/Ri during hepatic surgery.

## Background

Hepatic ischemia-reperfusion injury (I/Ri) is a serious and common adverse event during hepatic surgery that may ultimately lead to liver failure, systemic inflammatory response syndrome (SIRS) and even multiple organ failure syndrome (MOF) [1-4]. Central to hepatic I/Ri is the generation of reaction oxygen species (ROS) by activated Kupffer cells or neutrophils upon the reintroduction of molecular oxygen to ischemic tissues. This pathogenic event triggers a series of deleterious effects that include oxidative modification of lipids and proteins, induction of apoptosis in hepatocytes, release of pro-inflammatory cytokines, increased expression of adhesion molecules, and infiltration of leukocytes, which together leads to massive tissue destruction [5,6].

To ameliorate the severity of liver I/Ri, several therapeutic strategies are currently being pursued, including the inhibition of apoptosis by decreasing cellular metabolism using the gas hydrogen sulphide (H_2_S). Application of H_2_S has shown promising activity in various pre-clinical I/Ri and transplantation models, including kidney and liver [7,8]. A second interesting strategy is to inhibit mitochondrial calcium overload, e.g. with 2-ABP [9], and thus block the execution of mitochondrial apoptotic signaling [10]. A third particularly appealing strategy is the use of anti-oxidants that directly counteract the deleterious effects of ROS. In this respect, dietary anti-oxidative supplements such as rutin and L-arginine have shown beneficial effects on severity of hepatic I/Ri [11]. Moreover, carbon monoxide (CO) has raised particular therapeutic interest because of its potent anti-oxidant and anti-inflammatory activity.

CO is best known as an odorless and toxic gas which upon inhalation binds with high affinity to heme, thereby forming carboxyhemoglobin and severely impairing the respiratory system. However, CO is also produced by the protein heme oxygenase (HO) and as such functions as a potent endogenous antioxidant that counteracts toxic effects of ROS. HO-1 degrades heme into biliverdin, free iron, and CO [12] and is one of the most prominent lines of cellular defense against damage induced by I/Ri. Indeed, during oxidative stress the expression of the inducible form of HO, (HO-1) is strongly up-regulated in the liver [13].

Therapeutic up-regulation of CO tissue levels can be achieved via exogenous application of CO, for instance by direct inhalation of CO gas. In *ex vivo *isolated liver perfusion and liver transplantation models exogenously added CO yields potent cytoprotective effects [14-16]. Moreover, CO has anti-inflammatory activity e.g. by inhibiting the inflammatory response of donor Kupffer cells upon transplantation [17], activation of anti-inflammatory mitogen-activated protein kinase signaling [18], and up-regulation of the anti-inflammatory cytokine IL-10 [19]. Furthermore, CO inhibits the acquisition of a pro-adhesive phenotype of vascular endothelial cells [20].

Most studies on the role of CO in the amelioration of inflammatory responses have been performed by administration of gaseous CO [21]. However, the applicability of gaseous CO is limited by its toxic effect on cellular respiration. Therefore, therapeutic use of CO as a cytoprotective agent clearly requires a pharmaceutical formulation that allows for the selective delivery and/or local release of CO from a non-toxic pro-drug. In this respect, transitional metal carbonyl-based compounds are of particular interest because of their capability for controlled release of CO in biological systems [22]. These so-called CO-releasing molecules (CORMs) have been therapeutically tested in a variety of experimental inflammatory models with promising anti-inflammatory responses [12,23,24]. Indeed, treatment of septic mice with tricarbonyldichlororuthenium (II) dimer (CORM-2) strongly attenuated liver inflammation, as evidenced by a decrease in serum liver damage markers and a reduced influx of inflammatory cells[25]. In addition, CORM-2 was recently reported to improve outcome of ischemia reperfusion injury to the small bowel [26]. Importantly, CORM treatment is associated with low or minimal formation of carboxyhemoglobin and is therefore considered a safer alternative to CO gas inhalation [22].

Based on the above, we hypothesized that CORM-2 may ameliorate damage occurring during hepatic I/Ri. Here, we investigated this hypothesis in a rat model of experimentally induced hepatic I/Ri.

## Methods

### Rats and experimental procedure

All animal experiments were performed in accordance with the experimental protocol approved by the Committee for Research and Animal Ethics of Harbin Medical University. For the experiments, healthy male Wistar rats (n = 40; body weight, 230-250 g) were purchased from the Central Animal Facility of the First Clinical Hospital of Harbin Medical University (Harbin, China). Rats were housed in cages under standard animal care conditions and fed with rat chow ad libitum. All surgical procedures were performed under general anesthesia using sodium pentobarbital (50 mg/kg i.p.). Normothermic partial hepatic ischemia was induced by performing a midline laparotomy, exposing the liver hilum and subsequent clamping of portal structures to the left and median lobes with a microvascular clip, yielding ~70% hepatic ischemia [27]. The abdomen was covered during the ischemic period. After 60 min of partial hepatic ischemia, the clip was removed to initiate hepatic reperfusion and the abdominal cavity was closed with a 4-0 silk suture. Temperature was maintained at 37°C by a warming pad. Sham-operated rats underwent the same procedure without clamping the pedicle of the liver lobes.

Rats were randomly assigned into four groups with a sample size of 10. Sham group underwent a sham operation and received saline. I/R group underwent the hepatic I/R procedure and received saline. CORM-2 group underwent the same procedure and received 8 mg/kg of CORM-2 (Sigma-Aldrich, St. Louis, MO). iCORM-2 group underwent the same procedure and received iCORM-2 (8 mg/kg), which does not release CO. All treatments were administered prior to reperfusion. Rats were sacrificed by exsanguination at 6 hours post reperfusion upon which serum and liver samples were collected according to standard procedures. Rats in all experimental conditions survived the 6 hour reperfusion period.

### Serum transaminase determination

At the end of reperfusion, 5 ml of blood was collected from the caval vein in heparinized tubes. Samples were centrifuges at 800 g for 10 minutes at room temperature. Plasma was then used to evaluate the extent of hepatic injury by measuring serum levels of ALT and AST using an Automated Chemical Analyzer (7600; Hitachi, Tokyo, Japan). Values were expressed as units per liter (U/L).

### Liver histopathology score

Formalin-fixed liver samples were embedded in paraffin, sectioned into 5-μm thickness and stained with hematoxylin-eosin and microscopically inspected to assess inflammation and tissue damage. Histological examination of hepatic tissue damage was performed by two liver pathologists in a blinded fashion. Ten separated microscopic fields were scored on a scale from 0 to 3 as described before [28]. The severity of tissue damage was defined as: grade 0, minimal or no evidence of injury; grade 1, mild injury consisting of cytoplasmic vacuolation to focal nuclear pyknosis; grade 2, moderate to severe injury with extensive nuclear pyknosis, cytoplasmic hypereosinophilia, and loss of intercellular borders; grade 3, severe necrosis with disintegration of hepatic cords, hemorrhage, and neutrophil infiltration.

### Detection of apoptotic cells by terminal deoxyribonucleotide transferase (TdT)-mediated dUTP nick-end labeling (TUNEL) staining

TUNEL staining (Roche, Shanghai, China) was applied to paraffin-embedded 5-μm liver sections to detect DNA fragmentation as a measure for the number of apoptotic cells. Counterstaining was performed with 4',6-diamidine-2'-phenylindole dihydrochloride (DAPI) dye. Briefly, deparaffinized livers sections were incubated in permeabilization solution (0.1% TritonX-100 in 0.1% sodium citrate) for 2 min on ice, incubated with TUNEL reaction mixture for 60 min at 37°C in the dark and incubated for 4 min with DAPI dye in the dark. The numbers of apoptotic cells and total hepatic cells were counted in ten separated microscopic fields under 400× magnification. Numbers were then averaged and used to calculate the apoptosis index (AI) according to the previously reported formula: AI = (apoptotic cells/total hepatic cells) × 100% [29].

### Myeloperoxidase activity assay

Myeloperoxidase (MPO), an enzyme found predominantly in azurophilic granules of polymorphonuclear leukocytes, was measured as an index of neutrophil infiltration in the ischemic liver Briefly, liver tissue was homogenized, centrifuged at 12.000 g for 15 minutes at 4°C, after which MPO activity was measured using a commercial kit (NJJC Bio Inc., Nanjing, China) according to manufacturer's instructions. Absorbance was measured at 460 nm with a spectrophotometer. MPO activity was expressed as units per gram protein (U/g).

### Cytokine Analysis

Serum samples were obtained from rats 6 h after the I/R procedure, at the time-point of termination, and stored at -20°C until further analysis. Serum samples were analyzed for TNF-α and IL-6 levels using commercially available enzyme-linked immunosorbent assay (ELISA) kits (R&D Systems, Minneapolis, MN) according to manufacturer's instructions. Cytokine levels were expressed as picogram per ml (pg/ml).

### Western blot analysis

Liver whole cell homogenates and nuclear extracts were obtained by lysis of hepatic tissue with the Nuclear Extract Kit (Active Motif, Carlsbad, CA) according to manufacturer instructions. Protein aliquots (50 μg) were subsequently separated by SDS-PAGE on 5% or 10% acrylamide gels and proteins were electrotransferred to PVDF membrane. Protein levels were visualized by incubation with the following antibodies: bcl-2, caspase-3, ICAM-1, HO-1, NF-κB p65, β-actin, and Histone H1 (all from Santa Cruz Biotechnology, Inc. Santa Cruz, CA). Specific binding of antibodies was visualised using appropriate horseradish peroxidase-linked secondary antibody followed by detection of chemoluminescence using an enhanced chemoluminescence detection kit (Roche) according to manufacturer's instructions. Blots were stained with anti-β-actin or Histone H1 antibody to verify equal protein loading.

### Liver nuclear factor-kappa B (NF-κB) activation

Activation of the transcription factor NF-kB was measured using a commercially available ELISA kit (Trans-AM™ NF-kB p65 Transcription Factor Assay Kit, Active Motif, Carlsbad, CA) according to manufacturer's instructions. Nuclear protein extract was obtained using Nuclear Extract Kit (Active Motif) according to manufacturer's instruction. Subsequently, 15 μg nuclear protein extract was used to assay for NF-kB activation. Values were represented as OD450 nm.

### Statistical analysis

All values are expressed as the mean ± standard deviation (SD). Data were analyzed by one-way ANOVA followed by the Tukey-Kramer post test. For the semi-quantitative histopathological scores, statistical analysis was performed using the Kruskal-Wallis test followed by the Dunn's post test. P < 0.05 was considered to indicate statistical significance.

## Results

### CORM-2 attenuates hepatic ischemia reperfusion injury (I/Ri)

**Figure 1:**
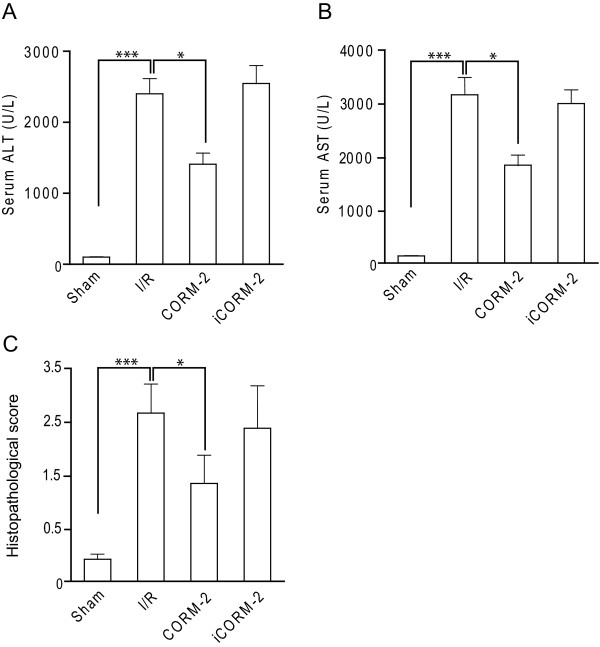
**CORM-2 attenuates hepatic ischemia reperfusion injury (I/Ri)**. Serum levels of **A **ALT and **B **AST of rats subjected to 60 min. of ischemia followed by 6 h of reperfusion. **C **Liver histology damage scoring of sham-operated rats, I/Ri rats, CORM-2 treated rats and iCORM-2 treated rats. *P < 0.05 vs. I/R group.

Endogenous CO, produced in response to stress by HO-1, is an important cellular tool to prevent cytotoxic and pro-inflammatory effects of ROS. Moreover, in various experimental animal models release of CO by carbon monoxide releasing molecules (CORM) has shown promising cytoprotective and anti-inflammatory activity [12,23,24]. Therefore, we hypothesized that the local release of CO using CORM-2 might be used for the prevention or reduction of ROS-mediated hepatic ischemia reperfusion injury (I/Ri). To test this hypothesis we treated rats subjected to I/R with CORM-2 at the time-point of reperfusion. In untreated I/R rats, the plasma levels of serum liver damage markers ALT and AST were significantly increased compared to sham-operated rats (Fig 1A and 1B, respectively; p < 0.001), indicative of significant liver hepatocyte injury and alterations in hepatic function by I/Ri. However, a single systemic administration of CORM-2 at the time-point of reperfusion significantly attenuated hepatic I/Ri as evidenced by a significant reduction in ALT and AST levels 6 hours post-reperfusion (Fig. 1A, p < 0.05 and B, p < 0.05). Semi-quantitative scoring of histopathological data confirmed that treatment with CORM-2 resulted in a significant reduction in liver injury score of I/Ri rats compared to untreated I/R rats (Fig. 1C, p < 0.05). Of note, although injury score was markedly improved by CORM-2 treatment, it was still elevated compared to sham-operated rats. Importantly, treatment with an inactive form of CORM-2 (iCORM-2), incapable of releasing CO, did not reduce liver I/Ri, indicating that release of CO is important for therapeutic activity (Fig. 1C). Taken together, these data clearly demonstrate that CO released by CORM-2 can ameliorate the negative effects of hepatic I/Ri.

### CORM-2 treatment inhibits apoptosis in hepatic I/Ri by up-regulation of Bcl-2

**Figure 2:**
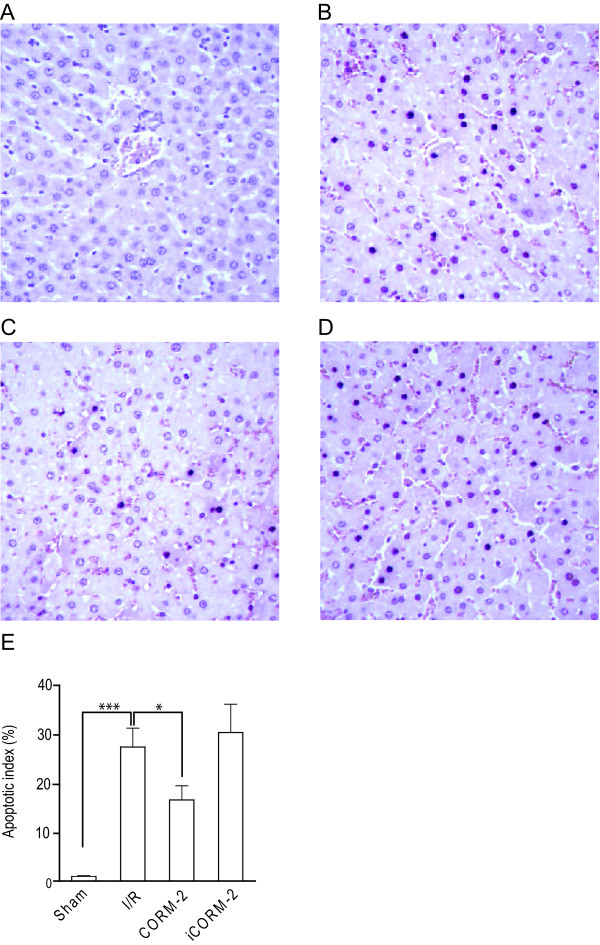
**CORM-2 treatment inhibits apoptosis in hepatic I/Ri**. Hepatocyte apoptosis was visualised by TUNEL staining in biopsies of **A **Sham-operated rats, **B **I/Ri-treated rats, **C **CORM-2 treated rats, **D **iCORM-2 treated rats. **E**. Graph of the apoptotic index in the various treatment groups. *P < 0.05 vs. I/R group.

**Figure 3:**
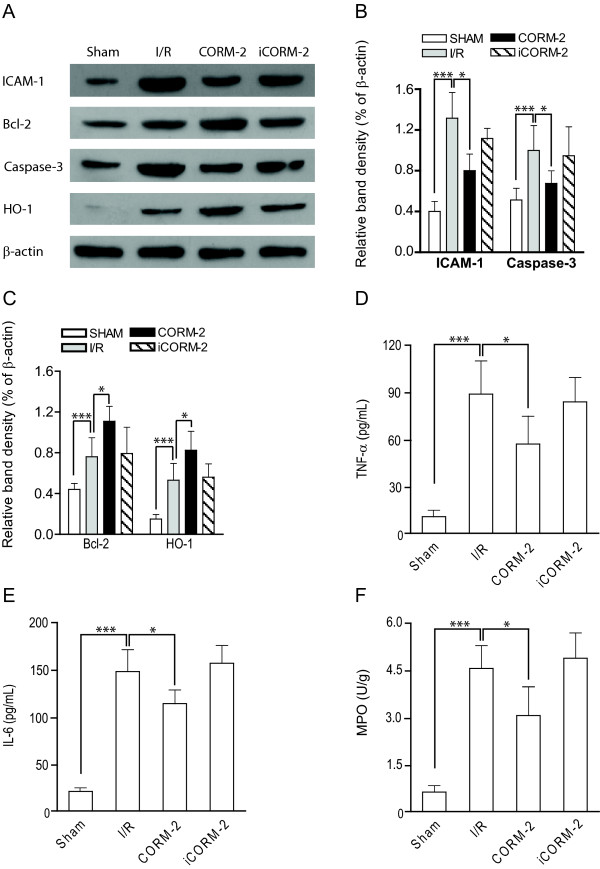
**CORM-2 modulates pro- and anti-apoptotic proteins and decreases neutrophil infiltration**. **A **Western blot analysis of the expression of ICAM-1, caspase-3, Bcl-2 and HO-1 in sham-operated, I/Ri operated, CORM-2 treated and iCORM-2 treated rats. **B **The relative expression levels of ICAM-1 and caspase-3 or **C **Bcl-2 and HO-1 were analysed with densitometry analysis in relation to β-actin. **D **Serum levels of TNF-α and **E **IL-6 of rats subjected to 60 min. of ischemia followed by 6 h of reperfusion. **F **Liver tissue MPO activity for each group was determined. *P < 0.05 vs. I/R group.

An important consequence of hepatic I/Ri is the loss of hepatocytes due to induction of apoptosis. Earlier studies have shown that inhalation of gaseous CO can attenuate apoptotic cell death in I/Ri models of the heart [30], lung, kidney [31], and small intestine[32]. Based on these well-established cytoprotective effects of CO, we assessed whether CORM-2 treatment reduced the extent of hepatocyte apoptosis in our rat hepatic I/Ri model using TUNEL staining. In non-ischemic livers of sham-operated rats only very few apoptotic cells were observed (Fig. 2A), whereas rats subjected to hepatic I/Ri had a dramatically increased number of apoptotic hepatocytes (Fig. 2B, p < 0.001). Importantly, treatment with CORM-2 markedly reduced the number of apoptotic hepatocytes (Fig. 2C, p < 0.05). In contrast, treatment of rats with iCORM-2 had no significant protective effect, with comparable numbers of TUNEL-stained hepatocytes in the non-treated I/R group and iCORM-2 group (Fig. 2D). Histological data were confirmed by counting apoptotic hepatocytes to obtain an apoptotic index. I/Ri significantly increased the apoptotic index compared to sham-operated rats (p < 0.001). Treatment with CORM-2 significantly reduced the apoptosis index compared to rats subjected to I/Ri (Fig. 2E, p < 0.05). Subsequent Western blot analysis of homogenized liver tissue confirmed that apoptosis was indeed inhibited by CORM-2, as evidenced by a reduction in the level of activation of effector caspase-3 (Fig. 3A and 3B, p < 0.05). Cleaved caspase-3 was strongly present in the I/Ri group and iCORM-2 treated group, whereas caspase-3 cleavage was markedly inhibited in CORM-2 treated rats.

The anti-apoptotic effect of CO has among others been attributed to up-regulation of anti-apoptotic members and down-regulation of pro-apoptotic members of the Bcl-2 family [31-33]. Indeed, we detected a strong up-regulation of the prototype anti-apoptotic protein Bcl-2 in CORM-2 treated rats when compared to either sham-operated rats (Fig. 3A, p < 0.001) or I/Ri rats (Fig. 3C, p < 0.05). Expression of Bcl-2 in I/Ri-rats was also increased in comparison to sham-operated rats, indicative of the initiation of the tissue homeostatic response. Together, these results indicate that CORM-2 exerts a protective effect on hepatocytes, at least in part, by up-regulation of Bcl-2 and concomitant inhibition of effector caspase activation.

### CORM-2 treatment inhibits production of pro-inflammatory cytokines

Inflammatory cytokines, such as TNF-α, are released by apoptotic and necrotic hepatocytes, vascular endothelial cells and/or Kupffer cells and are known to play key roles in the pathophysiology of hepatic I/Ri [34,35]. TNF-α is a major inducer of adhesion molecules (i.e. ICAM-1) on vascular endothelial cells and triggers the production of neutrophil attracting CXC chemokines [36,37]. Together, this leads to sinusoidal endothelial cell death and further hepatocyte damage. To determine whether the cytoprotective effect of CORM-2 was associated with a decrease in expression of this important pro-inflammatory mediator, we assessed serum levels of TNF-α. In line with literature, hepatic I/Ri strongly increased serum levels of TNF-α compared to base-line levels in sham-operated rats (Fig. 3D, p < 0.001). This increase in serum levels of TNF-α was significantly inhibited when rats were treated with CORM-2 (Fig. 3D, p < 0.05). In contrast, iCORM-2 did not affect serum levels of TNF-α after I/Ri (Fig. 3D).

Another important cytokine that is produced upon hepatic I/Ri is IL-6, which has long been assumed to play a pivotal role in liver tissue damage and as such is considered to be an important marker for the severity of tissue injury [38,39]. In our rat model, hepatic I/Ri induced high serum levels of IL-6 indicative of sever hepatic injury (Fig. 3E, p < 0.001). Of note, serum levels of IL-6 were significantly inhibited by treatment with CORM-2 (Fig. 3E, p < 0.05). Again, iCORM-2 did not have any effect (Fig. 3E). Thus, the induction of pro-inflammatory cytokines during hepatic I/Ri is markedly decreased by treatment with CORM-2.

### CORM-2 treatment prevents ICAM-1 expression and decreases neutrophil infiltration

To further clarify the mechanism of the protective effect of CORM-2 treatment, we assessed whether CORM-2 treatment also had an effect on neutrophil infiltration and activation. An important step in the tissue infiltration of leukocytes is the expression of adhesion molecules, such as ICAM-1, on vascular endothelial cells [40,41]. Indeed, down-regulation of ICAM-1 on vascular endothelial cells can attenuate hepatic I/Ri both *in vitro *and *in vivo *[42,43]. Several studies have shown that ICAM-1 is essential for leukocyte attachment and infiltration through endothelial cell lining in hepatic sinusoids [44,45]. Our data confirmed that expression of ICAM-1 in the liver was up-regulated as a result of hepatic I/Ri (Fig. 3A and 3B, p < 0.001). Moreover, administration of CORM-2, but not iCORM-2, markedly inhibited the ICAM-1 expression as induced by I/Ri (Fig. 3A and 3B, p < 0.05).

Next, we assessed whether this reduction in ICAM-1 expression was accompanied by a reduction in neutrophil infiltration. Neutrophil infiltration and activation is an important measure for tissue inflammation and can be quantified by determining tissue myeloperoxidase (MPO) activity [46]. MPO activity in the liver obtained from the I/Ri group was markedly increased compared with livers obtained from sham operated rats (Fig. 3F, p < 0.001). Consistent with the improvement in liver function, the activity of MPO significantly decreased upon CORM-2 administration (Fig. 3F, p < 0.05). In contrast, treatment with iCORM-2 did not affect tissue MPO activity. Thus, the expression of adhesion molecules and the subsequent tissue infiltration of leukocytes, in particular neutrophils, after hepatic I/Ri was effectively reduced by CORM-2 treatment.

### CORM-2 blocks pro-inflammatory NF-κB signaling in vivo

**Figure 4:**
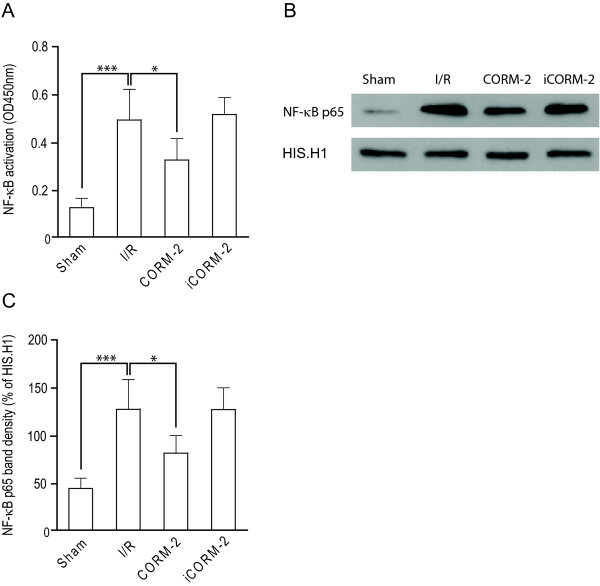
**CORM-2 blocks pro-inflammatory NF-κB signalling *in vivo***. NF-κB activation was determined using **A **ELISA-based TransAM NF-κB p65 kit and **B **visualised using western blot followed by **C **densitometry analysis.*P < 0.05 vs. I/R group.

The coordinated induction of hepatocyte apoptosis, the expression of pro-inflammatory cytokines, and the expression of vascular endothelial cell adhesion molecules results in the adhesion and migration of neutrophils and ultimately liver injury. All of these factors are known to be at least partly regulated by the transcription factor NF-κB. The active NF-κB unit involved in the pro-inflammatory response is the p50/p65 heterodimer, of which the p65 subunit provides the gene regulatory function. In order to evaluate whether the observed beneficial effects of CORM-2 was associated with a down-modulation of NF-κB activity, we assessed p65 subunit NF-κB DNA binding activity in hepatic nuclear extracts. I/Ri induced a significant increase in NF-κB p65 DNA binding activity (Fig. 4A, p < 0.001). This increase in I/Ri rats was significantly inhibited in hepatic nuclear extracts of I/Ri rats treated with CORM-2 (Fig. 4A, p < 0.05). In contrast, iCORM-2 had no effect on the activation of NF-κB. This effect of CORM-2 treatment on NF-κB was further confirmed by western blot detection of the presence of the p65 subunit in the nuclear fraction, which revealed a clear increase in protein levels of p65 subunit NF-κB in untreated I/R rats and iCORM-2 treated I/R rats (Fig. 4B). However, this translocation was inhibited by treatment with CORM-2 (Fig. 4B). Subsequent quantitation by densitometry revealed that CORM-2 treatment induced a significant reduction in nuclear translocation of the p65 subunit of NF-κB compared to untreated I/R rats and iCORM-2 treated I/R rats (Fig. 4C, p < 0.05). Taken together, these data indicate that the hepatoprotective effect of CORM-2 was in part due to down-regulation of the inflammatory mediators and inhibition of NF-κB activation.

### I/Ri-induced HO-1 expression is further augmented by CORM-2 treatment

The induction of HO-1 is an important cellular stress response that is induced by ischemia reperfusion. Therefore, we next assessed whether HO-1 expression was up-regulated by I/Ri and whether CORM-2 treatment had modulatory effects on this stress response. As anticipated, HO-1 expression in the liver obtained from I/Ri group was markedly increased compared to sham-operated animals. Interestingly, CORM-2 but not iCORM-2 treatment induced a further significant up-regulation of HO-1 (Fig. 3A and 3C, p < 0.05). These data confirm earlier studies, in which activation of the HO system by an HO-1 inducer or by HO-1 gene therapy displayed enhancement of hepatoprotection against warm and cold I/Ri in experimental animals [47,48]. Thus, CORM-2 treatment, by inducing local CO-production can help to increase HO-1 expression and thereby further augment the cytoprotective response upon I/Ri.

## Discussion

Endogenous CO produced by HO-1 is an important cellular protective measure to prevent cytotoxic and pro-inflammatory effects during reperfusion injury. Here we show that exogenous CO released by CO-releasing molecule 2 (CORM-2) can be applied to reduce hepatic ischemia reperfusion injury (I/Ri), a common adverse event during liver surgery that is characterized by hepatocellular death and inflammatory cell influx. In our model we demonstrated that CORM-2 treatment reduced the extent of apoptosis and ameliorated the pro-inflammatory stress response as evidenced by a reduction in the expression of pro-inflammatory cytokines, vascular endothelial adhesion molecule and a markedly reduced influx of leukocytes

Importantly, therapeutic application of CO inhalation is severely hampered by the deleterious effects on the respiratory system due to carboxyhemoglobin formation. For instance, inhalation of 500 ppm gaseous CO in humans resulted in a peak carboxyhemoglobin level of 7%, whereas in animal studies levels of up to 25% were detected. In contrast, treatment with CO-releasing molecules such as CORM-2 does not lead to a dramatic increase in carboxyhemoglobin. Indeed, treatment with CORM-2 at doses up to 20 μmol/kg (10,25 mg/kg) had no negative impact on oxy-haemoglobin saturation [48]. Thus, CORM-2 appears to be a potent inhibitor of negative effects of hepatic I/Ri, while at the same time having no appreciable negative effects on the respiratory system.

Both the cytoprotective and anti-inflammatory activity of CO appear to result, at least in part, from its ability to modulate the transcription factor NF-κB. For instance, CO-treatment of hepatocytes induces activation of NF-κB *in vitro *and *in vivo *[33], which renders these cells more resistant to apoptosis by up-regulating the anti-apoptotic protein Bcl-xL and down-regulating the pro-apoptotic protein Bax [49]. Similarly, we found a marked up-regulation of the anti-apoptotic protein Bcl-2 upon CORM-2 treatment. Since the balance between pro- and anti-apoptotic members of the Bcl-2 family is central to the control of the mitochondrial pathway of apoptosis, this increase in Bcl-2 expression is likely to inhibit execution of mitochondrial apoptosis. Of note, pre-treatment of LPS-stimulated human umbilical vein endothelial cells (HUVEC) with CO showed a reverse effect, namely inhibition of NF-κB activity [50]. As a result, CO-treated endothelial cells showed a reduced expression of adhesion molecules, which may reduce pro-inflammatory processes such as leukocyte adhesion and tissue infiltration of inflammatory cells. Thus, CO can have opposite effects on NF-κB signaling depending on the particular cell type involved. Further detailed investigation, using e.g. laser dissection microscopy may yield insight into the effect of CO on hepatocytes and hepatic vascular endothelium *in vivo*. However, from the above it is clear that these diverse effects on NF-κB cooperate to ameliorate cell damage and minimize inflammation.

In addition to NF-kB, protective effects of CO-released from CORM-2 may be related to the down-regulation of the iNOS/NO pathway in e.g. macrophages. *In vitro *treatment of LPS-stimulated macrophages with CO indeed prevented expression of iNOS and blocked the pro-adhesive phenotype [24,51]. Furthermore, treatment of I/Ri in a rat liver transplantation model using gaseous CO was partly attributable to down-regulation of iNOS/NO [16].

As anticipated, the induction of pro-inflammatory cytokines such as TNF-α during hepatic I/Ri is markedly decreased by treatment with CORM-2. Together with the accompanying decrease in expression of adhesion molecules these effects are likely accountable for the reduction in influx of inflammatory cells. The exact mechanism for down-regulation of TNF-α by CORM-2 treatment is still a matter of debate. Various reports have indicated that this effect might be attributable to direct CO-effects on vascular endothelium and circulating leukocytes. Indeed, CO has potent anti-inflammatory effects on LPS-stimulated HUVEC cells and macrophages [24,25,51]. Another possible contributing factor to the reduction in TNF-α level upon CORM-2 treatment is the rescue of hepatocytes from apoptosis. Apoptosis of hepatocytes is a universal feature of liver inflammation and is associated with the production of various inflammatory cytokines. Thus, the marked reduction in apoptotic hepatocytes upon CORM-2 treatment might contribute to the downplaying of the inflammatory response.

Of note, exogenous application of CORM-2 had an augmenting effect on the expression levels of HO-1, indicating that the exogenous addition of one of the reaction products of HO-1 has a positive feed forward effect on HO-1 expression. Since activation of the HO system by an HO-1 inducer or by HO-1 gene therapy enhances hepatoprotection against warm and cold I/Ri in experimental animals [46,47], HO-1 upregulation upon treatment with CORM-2 may contribute to the beneficial effects on severity of I/Ri. Indeed, products of the HO-1 enzyme such as bilirubin have well-documented cytoprotective and anti-oxidative activity. Further experiments, e.g. using specific HO-1 inhibitors such as zink protoporhyrin or OB-14 [52], may be used in conjunction with CORM-2 treatment to determine the relative contribution of these HO-1 products."

## Conclusion

In conclusion, exogenous CO as released by CORM-2 treatment has a cytoprotective effect during hepatic I/Ri, most likely mediated by the initial attenuation of apoptosis induction, followed by reduced expression of inflammatory mediators and adhesion molecules, and a concomitant decrease in neutrophil infiltration. Together with recent experimental evidence of beneficial effects of CORMs in kidney and small bowel I/Ri [26,53], these data clearly highlight the potential of CO-releasing molecules such as CORM-2 for the prevention or amelioration of I/Ri damage. Therefore, further pre-clinical investigation into the therapeutic applicability of controlled CO-release by CORM-2 for the prevention of I/Ri in hepatic surgery is warranted.

## Competing interests

The authors declare that they have no competing interests.

## Authors' contributions

YW carried out acquisition of data. PC carried out acquisition of data and performed statistical analysis. MB carried out acquisition and interpretation of data. WZ designed the study. EB analyzed and interpreted data and wrote the manuscript. WH: participated in coordination and writing. All authors read and approved the final manuscript.

## Pre-publication history

The pre-publication history for this paper can be accessed here:

http://www.biomedcentral.com/1471-230X/10/42/prepub
